# Histological Analysis of a Long Term Patent Subintimal Canal in the Superficial Femoral Artery

**DOI:** 10.1016/j.ejvsvf.2020.12.023

**Published:** 2020-12-29

**Authors:** Tormod Lund, Aud Svindland, Dag Bay, Jon O. Sundhagen, Jonny Hisdal, Tor Flørenes

**Affiliations:** a Department of Vascular Surgery, Division of Cardiovascular and Pulmonary Diseases, Oslo University Hospital, Oslo, Norway; b Faculty of Medicine, University of Oslo, Oslo, Norway; c Department of Pathology, Oslo University Hospital, Oslo, Norway; d Division of Radiology and Nuclear Medicine, Oslo University Hospital, Oslo, Norway

**Keywords:** Endothelialisation, Subintimal angioplasty, Superficial femoral artery

## Abstract

**Introduction:**

Subintimal angioplasty (SIA) was introduced in the late 1980s and is a supplement to bypass surgery. Adaptation of the technique has been hampered by high rates of early intervention to maintain patency, but the long term assisted patency is good.

**Report:**

The superficial femoral and popliteal artery containing a patent subintimal canal were explanted from a patient who died in the authors' ward. Histological analysis indicated that the lumen was created in the medial layer of the vessel wall. A collagen rich neointima and fragmented internal elastic lamina were observed, presumably as a result of activated smooth muscle cells. The luminal surface was partly covered by a single layer of CD31, von Willebrand factor, and partly CD144 positive cells. An early atherosclerotic lesion was observed distally in the subintimal canal.

**Discussion:**

Remodelling and neo-cellularisation of the vascular wall after SIA are described. Notably, hallmarks of early and late stage atherosclerotic disease were evident throughout the subintimal canal. These observations require confirmation in a larger number of specimens but underscore the need for surveillance after SIA.

## Highlights

• The subintimal canal was created in the medial layer of the vessel wall.
• Early and late stage atherosclerosis is observed throughout the subintimal canal.
• Neo-cellularisation of single layer luminal cells is apparent in the subintimal canal.
• The findings underscore the need for surveillance of subintimal canals.

## Introduction

Peripheral artery disease affects about 20% of the population aged > 60 years, of whom about 35% have symptoms. The disease is associated with the lowest quality of life of all symptomatic cardiovascular disease manifestations and is the costliest.^1^

**Figure 1:**
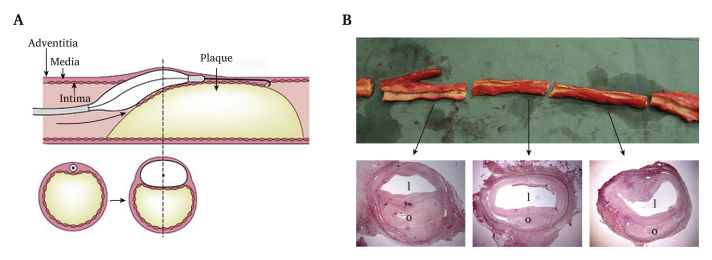
Subintimal angioplasty (SIA). (A) Schematic illustration: a guidewire is passed subintimally past the occlusion. A balloon is then introduced over the wire and inflated to create a new lumen. (B) The explanted superficial femoral and proximal popliteal artery. The distal segment of an occluded bypass is observed (left in the picture), the proximal part of the bypass was removed during surgical endarterectomy six years prior to explantation. Arrows point to representative, haematoxylin and eosin stained slides from the distal, middle and proximal parts of the subintimal canal. o = occluded lumen, l = new lumen created by SIA.

As a supplement to bypass surgery, recanalisation of occluded arteries using a subintimal angioplasty technique (SIA) was introduced by Bolia and co-workers in the late 1980s.^2^ In SIA, instead of advancing through a vessel occlusion intraluminally, a catheter and a little force is applied to enter the vessel wall subintimally, traverse the occlusion, and re-enter the vessel lumen distal to the occlusion. Subsequently, a balloon is introduced over the wire and inflated to produce a new lumen (Fig. 1A).

In this report, the superficial and popliteal artery containing a patent subintimal canal were explanted from a patient who died in the authors' ward. A detailed histological analysis of the subintimal canal is presented.

## Report

A 69 year old man presented with gradually worsening right calf pain on walking. Three years earlier he had undergone an above knee femoropopliteal bypass operation with a PTFE graft. The graft occluded, thrombolysis was initiated twice, but re-occlusion occurred shortly after both procedures despite antiplatelet therapy (aspirin 160 mg once daily) and warfarin. He had stopped smoking 12 years previously. Following months of exercise without improvement in symptoms, subintimal angioplasty (SIA) was performed via ipsilateral femoral access, and the entire superficial femoral artery was traversed with re-entry in the popliteal artery beyond the distal bypass graft anastomosis. A 6 mm balloon was inflated to create the new lumen (Fig. 1A). Completion angiography revealed a subintimal canal with good three vessel runoff below the knee (not shown). Warfarin was continued for three months after the procedure, and then stopped. Follow up with ultrasound one year later revealed stenosis in the proximal part of the subintimal canal, which was successfully treated with plain old balloon angioplasty (POBA). Then, following a symptom free period of a little less than a year, ultrasound examination again suggested stenosis, and POBA was performed, this time for a stenosis in the proximal and middle part of the canal. He was symptom free for the next two years, when the canal occluded acutely. Thrombolysis was performed, successfully, and the inflow tract of the canal was revised surgically shortly thereafter with removal of the proximal part of the PTFE graft and thrombo-endarterectomy of common femoral artery, the inflow tract of the subintimal canal. He was then symptom free for the following six years.

During those six years he had been taking aspirin and, for the last three years, a statin. He did not have, nor did he develop, diabetes. Then, at age 79, he was referred with an occluded bypass in his left leg. Thrombolysis was initiated, but the procedure was complicated by intracranial haemorrhage and the patient died. Consent was obtained from the patient's next of kin to remove the patient's right femoral and popliteal arteries, containing the patent subintimal canal, for analysis.

**Figure 2:**
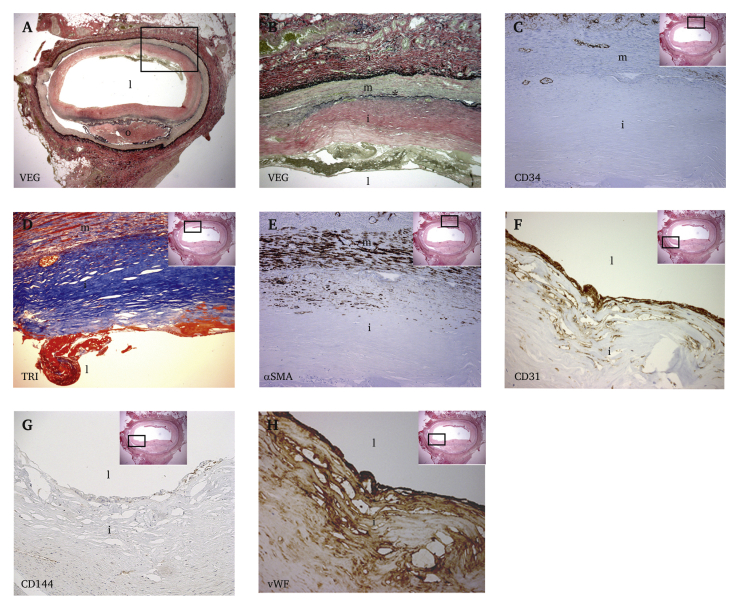
Immunohistochemistry of the middle part of the explanted superficial femoral artery (SFA). (A) Representative slide from the middle part of the SFA stained with Verhoeff's elastic stain (VEG), staining elastin black, ×12.5. A fibrin rich mural thrombus can be seen in grey/green colour in the upper luminal part. The rectangle depicts the area magnified in (B). (B) Thick elastic lamellae partly form an internal elastic lamina (IEL) (∗), ×100. The black rectangle in the inset image depicts the approximate area of magnification in (C – H). (C) Immunostaining for endothelial cells with CD34 shows marked adventitial vascularisation and a few vessels close to the IEL. Vascularisation of neointima is not observed, ×200. (D) Masson's trichrome stain (TRI), with collagen (blue) in neointima and muscle fibres (red) in the media. A part of the fibrin rich mural thrombus (red) can be seen luminally, ×200. (E) α-Smooth muscle actin (SMA) stained section shows smooth muscle cells and/or fibroblasts in the media and outermost in neointima, ×200. (F) Immune staining with the endothelial markers CD 31, ×400, (G) CD144, ×100 and (H) von Willebrand factor, ×400. o = occluded lumen, l = new lumen created by SIA, a = adventitia, m = medial layer, i = neointima, IEL = internal elastic lamina. (For interpretation of the references to colour in this figure legend, the reader is referred to the Web version of this article.)

**Figure 3:**
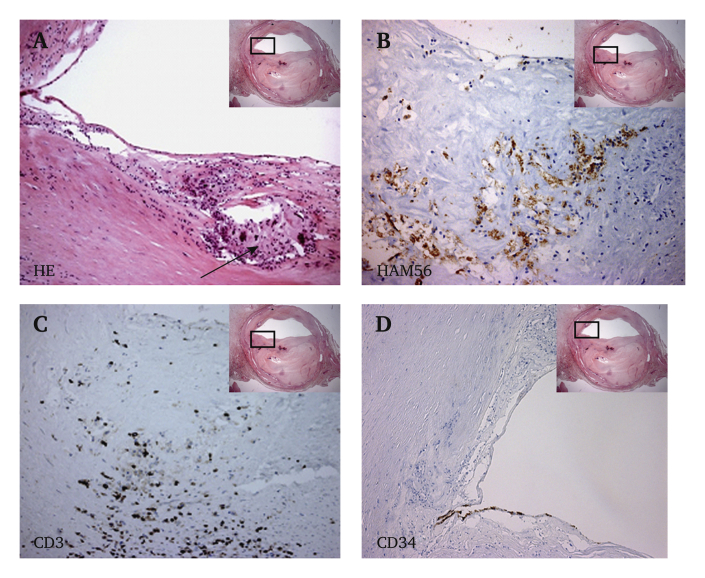
Immunohistochemistry of the distal part of the explanted superficial femoral artery (SFA). The black rectangle in the inset image depicts the approximate area of magnification. (A) Haematoxylin and eosin stained section shows an early atherosclerotic lesion with prominent foam cell aggregate (arrow) in neointima, ×100, and immunostained sections demonstrate infiltration of (B) macrophages (HAM-56), ×200, (C) T lymphocytes (CD3), ×200 and (D) a single layer of CD34 positive luminal cells, ×200.

The subintimal canal was approximately 30 cm in length. Histological examination suggested that the lumen was created in the medial layer of the vessel wall (Fig. 1B). Focusing on the middle part of the explanted artery, a thick, collagen rich neointima was observed (Fig. 2B and D). Further, a thin and fragmented internal elastic lamina (IEL) had formed circumferentially (Fig. 2A and B). Vasa vasorum, with single layer CD34 positive luminal cells were observed in the neointima close to the IEL (Fig. 2C). Alpha smooth muscle actin (αSMA) positive cells, probably smooth muscle cells or myofibroblasts, were found in the outermost part of the neointima and media (Fig. 2E). Stretches of the neointima were lined luminally by a single layer of CD31 positive cells (Fig. 2F). These luminal cells were von Willebrand (vWF) factor positive (Fig. 2H), some were CD144 (vascular endothelial (VE) cadherin) positive (Fig. 2G), but they were negative for endothelial nitric oxide synthase (eNOS), endoglin (CD105), and CD34 (data not shown). Patches of CD31 positive luminal cells were also found in the proximal part of the subintimal canal (data not shown). In the distal part of the subintimal canal there were foam cells, macrophages, and T cells (Fig. 3A–C), indicating an early atherosclerotic lesion. Here, covering the lesion, the single luminal cell layer was CD34 positive (Fig. 3D).

## Discussion

The main finding in this report is evidence of arterial wall remodelling and neo-cellularisation in a subintimal canal 10 years after it was created in the superficial femoral artery.

In SIA, a guide wire is used to traverse an occlusion blindly, where it follows the path of least resistance.^2^ It appears that this path, and the subsequent subintimal canal, is in the medial layer of the arterial wall (Fig. 1B). For surgeons who perform endarterectomies, this path corresponds to the plane of dissection from which an obstructing atherosclerotic plaque can be excised with ease. Indeed, medial muscle fibres are usually, if not always, part of endarterectomy specimens.^3^

During the SIA procedure, when the balloon is inflated to create a new lumen, profound activation of smooth muscle cells occurs. Subsequent increase in synthesis of extracellular matrix proteins, such as collagen and elastin, may be the result. The collagen rich neointima observed in the middle part of the subintimal canal (Fig. 2B and C) is probably a testament to this activation. However, it is noted that the innermost part of the neointima lacks α-SMA positive cells altogether (Fig. 2E). A similar histological picture has been observed in the human coronary artery in the late, fibrotic stage of atherosclerosis.^4^ Additionally, experimental studies have shown that smooth muscle cells within atherosclerotic plaques can undergo a phenotype switch and lose expression of α-SMA.^5^

A thin and fragmented internal elastic lamina (IEL), the inner “rubber band” of arteries mainly composed of elastin, can be observed in the middle part of the subintimal canal (Fig. 2B). It is known that the wall of arteries is subject to continuous remodelling through synthesis of enzymes that break down (e.g. the matrix metalloproteinases) and build up (e.g. collagen and elastin) the content of the wall. In atherosclerotic and inflamed arteries, it is not uncommon to observe fragmentation and sporadic duplication of the IEL.^6^ Taken together, the media and neointima in the middle part of the subintimal canal exhibit remodulation similar to that seen in the wall of atherosclerotic arteries.

Stretches of single layer luminal cells positive for CD31, vWF and partly VE-cadherin are observed in the middle part of the subintimal canal (Fig. 2F–H). Patches of single layer luminal CD31 positive cells are also observed, to a lesser extent, in the proximal parts of the canal (data not shown). The present results indicate that the subintimal canal is created within the collagen rich medial layer of the vessel wall, and recently collagen based grafts have been shown to support endothelialisation,^7^ however, it was not possible to show positive staining for eNOS in this case. It has been shown that in atherosclerosis, endothelial markers may be downregulated^8^ and the resting NO activity in muscular arteries may be low.^9^ The present authors believe that further studies, ideally including multiple specimens, are needed to clarify whether subintimal canals undergo neo-endothelialisation.

In the distal part of the canal, hallmarks of an early atherosclerotic lesion can be observed (Fig. 3A–C), with foam cells, infiltrating macrophages, and T cells. Atherosclerotic plaques tend to form at sites of flow disturbance, and the finding illustrates that subintimal angioplasty, and the remodulation and neo-cellularisation that may occur, does not prevent underlying atherosclerotic disease. Interestingly, the single luminal cell layer covering the lesion was CD34 positive (Fig. 3D). CD34 is considered a marker for endothelial cells of smaller blood vessels (e.g., vasa vasorum in Fig. 2C), but also immature endothelial cells.

Fifteen years ago, the present authors published results from 101 patients treated by SIA in the authors' department.^10^ It was reported that, after an initial period with a high prevalence of re-interventions, patency increased remarkably: of the 33 occlusions that occurred during a mean follow up of 41 months, 90% occurred within the first 15 months. It seems logical that the luminal surface of subintimal canals neo-cellularises or remodels in a fashion that, over time, renders it less thrombogenic. However, similar observations are made for prosthetic grafts in which a pseudo-intima consisting mostly of an acellular fibrinous lining is formed.

### Conclusion

Histological evidence is presented of arterial wall remodelling and neo-cellularisation in a subintimal canal 10 years after it was created in the superficial femoral artery. A large part of the remodelling resembles that of late stage atherosclerosis, but an early atherosclerotic lesion was observed in the distal part of the canal. There is evidence of neo-endothelialisation, but this must be confirmed. Whether or not these observations can explain why the patency of subintimal canals improves over time remains uncertain. The authors believe that it is safe to conclude that long term follow up of subintimal canals is warranted.
